# Clonal overlap and resistance profiles of multidrug-resistant *Klebsiella pneumoniae* in humans and domestic animals in Brazil: A One Health molecular epidemiology study

**DOI:** 10.14202/vetworld.2025.2012-2023

**Published:** 2025-07-22

**Authors:** Alessandra Tammy Hayakawa Ito de Sousa, Herica Makino, Marco Túlio dos Santos Costa, Stefano Luis Cândido, Kaio Lierlyson Teles Gomes, Cristiane Silva Chitarra, Marco Andrey Pepato, Francisco Kennedy Scofoni Faleiros de Azevedo, Francisco Jose Dutra Souto, Arleana Bom Parto Ferreira de Almeida, Valeria Régia Franco Sousa, Luciano Nakazato, Valéria Dutra

**Affiliations:** 1 Microbiology Laboratory, Veterinary Hospital of the Federal University of Mato Gross - UFMT, Cuiabá, Mato Grosso, Brazil; 2 Department of Clinical Medicine, Júlio Muller University Hospital of the Federal University of Mato Grosso - UFMT, Cuiabá, Mato Grosso, Brazil; 3 Medical Clinic at the Veterinary Hospital of the Federal University of Mato Grosso - UFMT, Cuiabá, Mato Grosso, Brazil

**Keywords:** *bla*_KPC-2_, *bla*_NDM_, Brazil, multidrug-resistant *Klebsiella pneumonia*, multilocus sequence typing, one health, virulence genes, zoonotic transmission

## Abstract

**Background and Aim:**

The global rise of multidrug-resistant (MDR) *Klebsiella pneumoniae* poses a serious threat to human and animal health. Close proximity between humans and domestic animals may facilitate zoonotic transmission of MDR strains, underscoring the need for integrated surveillance strategies. This study aimed to investigate the genetic diversity, resistance mechanisms, and virulence gene profiles of *K. pneumoniae* isolates from domestic animals and humans in Mato Grosso, Brazil, within the One Health framework.

**Materials and Methods:**

A total of 48 clinical isolates (33 from animals and 15 from humans) were analyzed. Identification was confirmed through 16S ribosomal RNA sequencing. Antimicrobial susceptibility was tested using disk diffusion (animal isolates) and minimum inhibitory concentration (human isolates). Resistance (*bla*_kpc-2_ and *bla*_NDM_) and virulence genes (*entB*, *fimH*, *wabG*, *ugE*, etc.) were detected through polymerase chain reaction. Multilocus sequence typing (MLST) was performed on seven housekeeping genes, and sequence types (STs) were assigned using the Pasteur Institute database (Paris, France).

**Results:**

MDR phenotypes were found in 70.83% (34/48) of isolates – 78.78% of animal and 53% of human samples. Virulence genes were present in 77.08% of isolates; *entB* was the most prevalent (60.61%). The *bla*_kpc-2_ gene was found in three human isolates, and *bla*_NDM_ was found in one human and one bovine isolate. MLST revealed 39 STs, including 9 novel ones. Clonal complexes (CC)258 (human), CC15 (animal), and CC147 (both species) indicated potential interspecies transmission.

**Conclusion:**

This study provides the first comprehensive molecular epidemiological snapshot of *K. pneumoniae* in domestic animals and humans in Mato Grosso. The discovery of shared clonal complexes and high MDR rates demands urgent cross-sectoral surveillance and control strategies under the One Health approach.

## INTRODUCTION

*Klebsiella pneumoniae* is an opportunistic pathogen commonly isolated from the mucosal surfaces of humans and animals, as well as from environmental sources such as water, soil, vegetation, and food [1]. Multidrug-resistant (MDR) strains of *K. pneumoniae* pose a significant public health concern, particularly under the One Health paradigm, due to their ability to circulate across human, animal, and environmental interfaces [2]. The pathogenicity of *K. pneumoniae* is driven by a range of virulence factors that facilitate adherence to host tissues, iron acquisition, and imm-une evasion. These virulence traits are primarily mediated by structural components such as capsules, lipopolysaccharides, and fimbriae, which not only enhance adaptability but also contribute to antimicrobial resistance by supporting enzyme production that neutralizes therapeutic agents [3, 4].

Antimicrobial resistance further amplifies the threat posed by *K. pneumoniae*, frequently leading to difficult-to-treat infections and hospital outbreaks. The severity of infection is often associated with the multidrug resistance profile of the isolate, a problem exacerbated by the overuse and misuse of antibiotics in both clinical and veterinary settings [5]. Of significant concern is the emergence of carbapenem-resistant strains, particularly those producing *K. pneumoniae* carbapenemase (KPC), which have been strongly linked to increased mortality rates [6, 7]. Another resistance mechanism of growing concern is the *bla*_NDM_ gene, which encodes New Delhi metallo-β-lactamase and contributes to the rapid global dissemination of resistant clones [7].

To monitor and control the spread of these high-risk strains, molecular epidemiological tools such as multilocus sequence typing (MLST) have become indispensable. MLST offers high-resolution genotyping by sequencing conserved regions of multiple housekeeping genes, enabling precise characterization of clonal lineages. This technique is particularly useful for identifying zoonotic transmission pathways and tracing interspecies dissemination of resistant clones. Furthermore, MLST data contribute to international databases, thereby strengthening global surveillance efforts and informing public health interventions at both regional and international levels [8].

Despite the growing recognition of *K. pneumoniae* as a critical MDR pathogen with zoonotic potential, there remains a significant lack of data on its molecular epidemiology in underrepresented regions such as Mato Grosso, Brazil. Most available studies have concentrated on human clinical settings, with limited integration of data from veterinary and environmental sources, thereby hindering a comprehensive understanding of transmission dynamics. Furthermore, while global MLST databases continue to expand, there is still a paucity of genotypic data linking animal and human isolates from the same geographical area. This hampers our ability to detect interspecies clonal dissemination and the emergence of high-risk clones, such as those carrying carbapenemase genes such as *bla*_KPC-2_ and *bla*_NDM_. In addition, few studies in Brazil have concurrently evaluated both virulence gene profiles and antimicrobial resistance mechanisms within a One Health framework. The lack of such integrated surveillance limits the development of effective, locally tailored intervention strategies. Therefore, filling this gap is essential to inform public health policies, antimicrobial stewardship programs, and veterinary practices in both regional and global contexts.

In response to these gaps, the present study aimed to conduct a comprehensive molecular characterization of *K. pneumoniae* isolates collected from both domestic animals and humans in the state of Mato Grosso, Brazil. Specifically, the study sought to (i) assess the prevalence of multidrug resistance and identify phenotypic resistance profiles, (ii) detect the presence of key resistance determinants, particularly *bla*_KPC-2_ and *bla*_NDM_, (iii) investigate the distribution of virulence-associated genes, and (iv) characterize the clonal structure of the isolates using MLST. By comparing genotypic patterns and resistance markers across animal and human isolates, the study aimed to uncover potential interspecies transmission pathways and identify emerging high-risk clones. Ultimately, these findings are intended to provide a foundational dataset for regional epidemiological monitoring and contribute to the broader global surveillance of MDR *K. pneumoniae* within the One Health framework.

## MATERIALS AND METHODS

### Ethical approval and Informed consent

Ethical approval was obtained from the insti- tutional review boards of both participating hospitals. The study was approved by the Ethics and Research Committee on the Use of Animals of the Universidade Federal de Mato Grosso (UFMT) (protocol number: 23108.236834/2017-13) and by the Research Ethics Committee of Plataforma Brasil at the Hospital Universitário Júlio Muller, also affiliated with UFMT (protocol number: 82549918.1.0000.5541). Verbal informed consent was obtained from all human participants as well as from the owners of the animals included in the study.

### Study period and location

Clinical isolates were obtained from specimens collected between January 2016 and December 2017 at the participating hospitals, including the Veterinary Hospital at the Federal University of Mato Grosso and the Júlio Muller University Hospital.

### Sample collection and bacterial isolation

Clinical isolates were obtained from specimens collected at veterinary and human hospitals and processed in the microbiology laboratories of the Veterinary Hospital of the Federal University of Mato Grosso and the Júlio Muller University Hospital. Clinical specimens were systematically collected from defined anatomical sites in domestic animals and human patients admitted to veterinary and medical hospitals across the state of Mato Grosso. Although the samples were collected during an earlier period, the scarcity of data from this region underscores the significance of publishing these findings to bridge existing knowledge gaps and inform integrated One Health public health strategies.

### Collection, bacterial isolation, and identification

Animal clinical specimens were plated on 5% sheep blood agar (Sigma-Aldrich, Darmstadt, Germany) and MacConkey agar (Neogen Corporation, São Paulo, Brazil) and incubated aerobically at 37°C for 24–48 h. Suspected *K. pneumoniae* colonies were confirmed using biochemical tests, following the protocol outlined by Quinn *et al*. [9]. Human isolates were identified as *K. pneumoniae* using the VITEK 2 automated system (bioMérieux, Marcy l’Étoile, France).

### Antimicrobial susceptibility testing (AST)

AST was conducted using the Kirby–Bauer disk diffusion method [9], Inhibition zone diameters were interpreted using CLSI and Brazilian Committee on AST (BrCAST) standards [10, 11]. The American Type Culture Collection *K. pneumoniae* 13883 strain was used as a positive control to ensure assay accuracy and data reliability. Resistance profiles were assessed using 12 antibiotics spanning key antimicrobial classes: Penicillins (amoxicillin–clavulanate), cephalosporins (cephalexin), carbapenems (imipenem, meropenem), aminoglycosides (amikacin, gentamicin), quinolones (ciprofloxacin), phenicols (chloramphenicol), tetracy-clines (doxycycline), nitrofurans (nitrofurantoin), and sulfonamides (with and without trimethoprim) (Bio-Rad Brazil, Rio de Janeiro, Brazil). Resistance classification followed CLSI and BrCAST standards [11–13].

Minimum inhibitory concentrations (MICs) for human isolates were determined using the BacT/ALERT 3D (bioMérieux) and VITEK 2 Compact systems (bioMérieux) as per the manufacturer’s guidelines. The use of different AST methodologies reflected disparities in laboratory resources and infrastructure between the veterinary and human hospitals. All MIC interpretations strictly adhered to CLSI [12] criteria. Testing encom-passed seven antimicrobial classes comprising 16 antibiotics (BD BBL Sensi-Disc, Becton Dickinson, USA), including penicillins (ampicillin with sulbactam and piperacillin with tazobactam), cephalosporins (cefepime, cefoxitin, ceftazidime, ceftriaxone, cefuroxime, and cefuroxime axetil), carbapenems (ertapenem, imipenem, and meropenem), aminoglycosides (amikacin and gentamicin), quinolones (ciprofloxacin), polymyxin (colistin), and glycylcycline (tigecycline) [12]. Isolates were classified based on the MDR criteria defined by Magiorakos *et al*. [14], where resistance to one or more agents in at least three antimicrobial categories constitutes MDR status.

### DNA extraction and molecular identification

Genomic DNA was extracted by inoculating bacterial colonies into brain–heart infusion broth and then incubating them overnight at 37°C with agitation. After centrifugation, the resulting pellet was resuspended in 1 mL of lysis buffer and processed through the phenol-chloroform method described by Sambrook and Russell [15]. The extracted DNA was resuspended in 50 μL of ultrapure water and stored at −20°C for further analysis. DNA integrity and purity were verified through agarose gel electrophoresis. The extracted DNA underwent 16S *ribosomal RNA* gene amplification through polymerase chain reaction (PCR) using primers 27F (AGAGTTTGATCCTGGCTCAG) [16] and 1492R (GGTTACCTTGTTACGACT) [17]. The PCR products were purified using the illustra ExoProStar 1-STEP Kit (GE Healthcare Life Sciences, Cytiva, Marlborough, MA, USA) and then sequenced with the BigDye Terminator Ready Reaction Cycle Sequencing kit (Applied Biosystems, Foster City, CA, USA) on an ABI 3500 Genetic Analyzer (Applied Biosystems, a brand of Thermo Fisher Scientific headquartered in Waltham, Massachusetts, USA). Seq-uences were compared with GenBank (National Center for Biotechnology Information – NCBI, a division of the U.S. National Library of Medicine at the National Institutes of Health [NIH], headquartered in Bethesda, Maryland, USA) entries using BLAST (http://www.ncbi.nlm.nih.gov/BLAST) (National Center for Biotechnology Information (EUA). BLAST: Basic Local Alignment Search Tool [software online]. Bethesda [MD]: U.S. National Library of Medicine), and when applicable, deposited. Accession numbers and isolate metadata are available in the supplementary table. Chimeric sequences were screened and removed using DECIPHER software (Biomatters Ltd. (New Zealand). DECIPHER: sequence analysis software [software online]. Auckland [NZ]: Biomatters) to ensure data quality.

### Detection of virulence and resistance genes

PCR was used to detect seven virulence genes and the resistance genes *bla*_KPC-2_ and *bla*_NDM_, using primer sequences listed in Table 1 [18–23]. Previously characterized positive control samples harboring the target resistance genes were used to validate the PCR assays. These controls were also sequenced to confirm the presence of primer-specific nucleotide regions. PCR was conducted on a MyCycler™ Thermal Cycler (Bio-Rad, Hercules, CA, USA) in 25 μL reactions containing 25 ng of DNA, 1 U Taq polymerase (Sigma, MilliporeSigma, St. Louis, MO, USA), 1 mM deoxynucleoside triphosphates, 15 mM MgCl_2_, 1× PCR buffer (comprising 200 mM Tris-HCl, pH 8.4, and 500 mM KCl), and 20 pmol of each primer. The amplification protocol included an initial denaturation at 94°C for 5 min, followed by 35 cycles of denaturation at 94°C for 2 min, annealing at 50°C for 1 min, and extension at 72°C for 1 min, concluding with a final extension at 72°C for 5 min. Amplification of *bla*_KPC-2_ and *bla*_NDM_ genes followed similar cycling conditions with primer-specific annealing and extension settings. PCR products were visualized on 1.5% agarose gels stained with GelRed (Biotium, Fremont, CA, USA), and images were captured using the ChemiDoc XRS system and Image Lab software (Bio-Rad Laboratories, Hercules, CA, USA). Real-time PCR for *bla*_NDM_ detection was carried out on the QuantStudio 5 system (Thermo Fisher Scientific, USA) using TaqMan Fast Advanced Master Mix (Thermo Fisher Scientific), with thermal cycling consisting of an initial denaturation at 95°C for 20 s, followed by 40 cycles of 95°C for 3 s and 60°C for 30 s, as described by Feng *et al*. [23].

**Table 1:** *Primers* used to detect virulence genes and the *bla*_KPC-2_ resistance gene in *K. pneumoniae* isolates.

Gene	Function/location	Sequence (5’–3’)	Product (pb)	Reference
*fimH*	Production of fimbrial adhesions	GCT CTG GCC GAT AC YAC S AC GG GC RWA R TAA CG YGCC TGG AAC GG	423	[18]
*mrkD*	Production of fimbrial adhesions	TATYGKCTT AAT GGC GCT GG TAA TCG TAC GTC AGG TTA AAG AYC	945	[18]
*wabG*	Lipopolysaccharide Biosynthesis	ACC ATC GGC CAT TTG ATA GA CGG ACT GGC AGA TCC ATA TC	683	[18]
*ugE*	Lipopolysaccharide Biosynthesis	TCT TCA CGC CTT CCT TCA CT GAT CAT CCG GTC TCC CTG TA	535	[19]
*entB*	Transport of siderophore	CGC CCA GCC GAA AGA GCA GA CAT CGG CAC CGA ATC CAG AC	508	[20]
*KfuBC*	Iron transportation	GAA GTG ACG CTG TTT CTG GC TTT CGT GTG GCC AGT GAC TC	797	[21]
*bla*_KPC2_	Carbapenemase enzyme	TGTCACTGTATCGCCGTC CTCAGTGCTCTACAGAAAAACC	1011	[22]
*NDM*	New Delhi metallo-β-lactamase	CAGCAACCGCGCCCAACTTTGGCCCGCTCAAGG TTGATCAGGCAGCCACCAAAAGCGATGTCGG FAM-TTTTACCCCGGCCCCGGCCACACCAGTGACAA-BHQ1	121	[23]

### MLST genotyping and sequence analysis

MLST was performed by amplifying seven housekeeping genes (*gapA*, *infB*, *mdh*, *pgi*, *phoE*, *rpoB*, and *tonB*), as specified by the Pasteur Institute’s MLST protocol. The resulting sequences were compared to global MLST databases to determine sequence types (STs), and the GoeBURST algorithm was used to group STs into clonal complexes (CCs). Novel alleles and STs were submitted to the Pasteur Institute MLST database (http://bigsdb.pasteur.fr/klebsiella/klebsiella.html) [24].

### Statistical and bioinformatics analysis

Bioinformatic analysis, including multiple sequence alignment and polymorphism evaluation, was conducted using Clustal Omega (European Molecular Biology Laboratory, United Kingdom). Population genetic metrics such as nucleotide diversity (π) were calculated to assess genetic variation among isolates. Descriptive statistical analysis was carried out using R: A language and environment for statistical computing (version 4.3.1) [software online] (Vienna, Austria). Data were organized into tables and visualized using graphs to facilitate interpretation. Although no infer-ential statistical tests were applied, a significance threshold of p < 0.05 was maintained. Data were further standardized and reanalyzed using the Pasteur Institute MLST database to ensure consistency. Isolates identified as members of the *K. pneumoniae* complex but not specifically as *K. pneumoniae* were excluded, maintaining data integrity for global comparison and epidemiological interpretation.

## RESULTS

### Origin of isolates

Of the 48 *K. pneumoniae* isolates analyzed, 69% (33/48) originated from domestic animals and 31% (15/48) from human sources (Table 2).

**Table 2:** Distribution of *Klebsiella pneumoniae* isolates by host category—domestic animals versus humans—in Mato Grosso (Midwest Brazil), 2016–2017.

Host category	Host	Total
Domestic animals (n = 33)	Canine	15
	Equine	8
	Feline	5
	Bovines	4
	Pig	1
Humans (n = 15)	Human	15
Total		48

### Anatomical distribution of samples

Samples were obtained from 15 different anatomical sites. The most frequent sources included urine (34%, 16/48), rectal swabs (15%, 7/48), ear swabs (11%, 5/48), and lung lavage fluid (11%, 5/48) (Table 3).

**Table 3:** Absolute frequency and percentage of isolates site of 48 *Klebsiella pneumoniae*, Mato Grosso.

Sites/samples	Absolute frequency	Percentage
Urine	16	34
Rectal swab	7	15
Ear swab	5	11
Tracheobronchial lavage	3	6
Gastric contents	2	4
Eye swab	2	4
Lung	2	4
Liver	2	4
Catheter point	2	4
Blood	2	4
Interdigital secretion swab	1	2
Milk	1	2
Chest fluid	1	2
Abdominal cavity fluid	1	2
Gastric ulcer secretion	1	2
Total	48	100

### Antimicrobial resistance profiles

Disk diffusion testing revealed high antimicrobial resistance rates, consistent with CLSI and BrCAST standards [11–13]. Among animal isolates, nitrofurantoin and sulfonamides showed the highest resistance, each affecting 88% (29/33) of samples. The lowest resistance rates were noted for imipenem (0%, 0/33), meropenem (1%, 1/33), and amikacin (2%, 1/33) (Table 4). MDR phenotypes were identified in 78.78% (26/33) of animal isolates and 53% (8/15) of human isolates, yielding an overall MDR rate of 70.83% (34/48).

**Table 4:** Antimicrobial resistance profile of *Klebsiella pneumoniae* isolates using agar disk diffusion in animal isolates and the automated VITEK2 method (broth microdilution) in human isolates.

Antimicrobial categories	Resistance* (%)		

	Agent	Domestic animals (%) (n = 33)	Humans (%) (n = 15)
Penicillins β-lactams	AMC	42 (14/33)	NR
	PIT	NR	60 (9/15)
	AMP/sub	NR	60 (9/15)
Cephalosporins β-lactams	CFE	31 (12/33)	NR
	CPM	NR	60 (9/15)
	CFO	NR	53 (08/15)
	CAZ	NR	60 (09/15)
	CRO	NR	60 (9/15)
	CRX	NR	60 (9/15)
	CRX axetil	NR	60 (9/15)
Carbapenems β-lactams	MPM	1 (4/33)	53 (08/15)
	IPM	0 (0/33)	53 (08/15)
	ERT	NR	53 (08/15)
Aminoglycosides	AMI	21 (7/33)	13 (02/15)
	GEN	48 (16/33)	47 (07/15)
Quinolones	CIP	48 (16/33)	47 (07/15)
Fenicol	CLO	45 (15/33)	NR
Polymyxin	COL	NR	33 (05/15)
Tetracyclines	DOX	45 (15/33)	NR
Nitrofurans	NIT	88 (29/33)	NR
Sulfonamides	SUL	88 (29/33)	NR
	SUT	42 (14/33)	NR
Glycylcyclines	TIG	NR	60 (9/15)

Legend: AMC=Amoxicillin+clavulanic acid, AMI=Amikacin, AMP/sub=Ampicillin with sulbactam, CAZ=Ceftazidime, CFE=Cephalexin, CFO=Cefoxitin, CIP=Ciprofloxacin, CLO=Chloramphenicol, COL=Colistin (polymyxin E), CTF=Ceftiofur, CPM=Cefepime, CRO=Ceftriaxone, CRX=Cefuroxime, DOX=Doxycycline, ENO=Enrofloxacin, ERT=Ertapenem, GEN=Gentamicin, IPM=Imipenem, MBF=Marbofloxacin, MTZ=Metronidazole, MPM=Meropenem, NEO=Neomycin, NIT=Nitrofurantoin, PIT=Piperacillin/tazobactam, SUL=Sulfonamide, SUT=Sulfonamide+trimethoprim, TIG=Tigecycline, NR=Not realized.

* Samples classified as intermediately resistant were regrouped with those classified as resistant Human isolates exhibited lower resistance to amikacin (13%, 02/15) and colistin (33%, 05/15), with 50% of the isolates classified as MDR. Overall, 86% (41/48) of the isolates were MDR

### Virulence and resistance gene distribution

Virulence gene screening showed that 77.08% (37/48) of isolates harbored at least one virulence factor. In animal isolates, *entB* was the most prevalent (60.61%, 20/33), followed by *wabG* and *fimH* (both 48.48%, 16/33). Among human isolates, the prevalence of virulence genes was as follows: *ugE* (60%, 9/15), *entB* (46.67%, 7/15), *fimH* (33.33%, 5/15), and *wabG* (33.33%, 5/15), regardless of sample site. Regarding resistance genes, *bla*_KPC-2_ was detected in 3 out of 15 (20%) human isolates but was absent in all animal-derived samples. The *bla*_NDM_ gene was found in one human rectal swab isolate (6.66%, 1/15) and one bovine ocular swab isolate (3.03%, 1/33) (Supplementary material).

### MLST results

MLST analysis identified 39 distinct STs, including 9 novel ones (ST4527–ST4535). These STs were grouped into 32 CCs and six singletons (Table 5). Clonal complex CC258 was the most frequent in human isolates (27%, 4/15), while CC15 appeared in 9% (3/33) of animal isolates. CC147 was the only clonal complex found in both humans (ST392) and animals (ST4530, canine origin), indicating potential interspecies clonal overlap.

**Table 5:** *Klebsiella pneumoniae* isolates with the species, isolation site, ST and CC of the 49 isolates.

Species	Isolation site	ST	CC
*Canis lupus familiaris*	Ear swab	35	35
*C. lupus familiaris*	Ear swab	4529	993
*C. lupus familiaris*	Ear swab	1393	1393
*C. lupus familiaris*	Ear swab	4546	*Singletons*
*C. lupus familiaris*	Urine	983	983
*C. lupus familiaris*	Urine	15	15
*C. lupus familiaris*	Urine	4530	147
*C. lupus familiaris*	Urine	15	15
*C. lupus familiaris*	Urine	873	873
*C. lupus familiaris*	Urine	1824	1059
*C. lupus familiaris*	Urine	307	307
*C. lupus familiaris*	Interdigital secretion swab	788	788
*C. lupus familiaris*	Eye swab	2474	2474
*C. lupus familiaris*	Chest fluid	889	889
*C. lupus familiaris*	Rectal swab	4527	*Singletons*
*Felis catus*	Urine	307	307
*F. catus*	Urine	1110	152
*F. catus*	Ear swab	20	20
*F. catus*	Abdominal cavity fluid	4531	101
*F. catus*	Rectal swab	567	567
*Sus scrofa domesticus*	Lung	188	188
*Bos taurus*	Milk	231	231
*B. taurus*	Rectal swab	289	289
*B. taurus*	Eye swab	3494	*Singletons*
*B. taurus*	Liver	266	1401
*Equus caballus*	Rectal swab	1089	*Singletons*
*E. caballus*	Rectal swab	1089	*Singletons*
*E. caballus*	Gastric contents	534	534
*E. caballus*	Gastric contents	534	534
*E. caballus*	Tracheobronchial lavage	3883	*Singletons*
*E. caballus*	Liver	2035	3849
*E. caballus*	Urine	15	15
*E. caballus*	Lung	534	534
*Homo sapiens sapiens*	Blood	11	258
*H. sapiens sapiens*	Blood	634	1373
*H. sapiens sapiens*	Rectal swab	392	147
*H. sapiens sapiens*	Rectal swab	392	147
*H. sapiens sapiens*	Urine	437	258
*H. sapiens sapiens*	Tracheobronchial lavage	11	258
*H. sapiens sapiens*	Urine	12	12
*H. sapiens sapiens*	Catheter tip	11	258
*H. sapiens sapiens*	Urine	4532	585
*H. sapiens sapiens*	Catheter tip	323	323
*H. sapiens sapiens*	Urine	4533	524
*H. sapiens sapiens*	Urine	4534	17
*H. sapiens sapiens*	Urine	70	70
*H. sapiens sapiens*	Sputum	60	60
*H. sapiens sapiens*	Urine	12	12
*H. sapiens sapiens*	Ulcer secretion	4535	70

CC=Clonal complexes, ST=Sequence type

### Genetic diversity analysis

**Figure 1:**
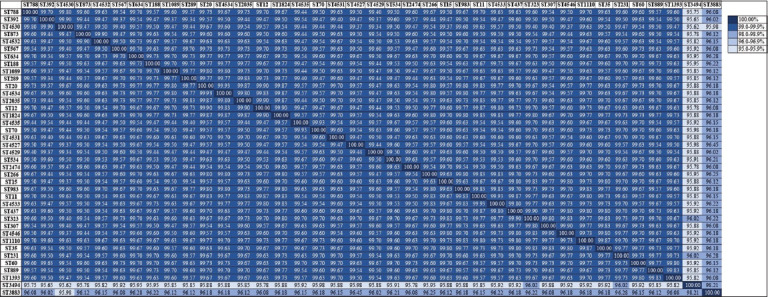
Percent identity and divergence score matrix of the seven concatenated gene nucleotide sequence type of *Klebsiella pneumoniae* from animals and humans.

The concatenated gene sequence analysis revealed no insertions, deletions, or tetra-allelic single-nucleotide polymorphisms. Pairwise sequence identity ranged from 95.62% to 100%. Among the 3,012 nucleotide positions assessed, 188 sites (6.24%) were polymorphic. The nucleotide diversity index (π) was calculated to be 0.01843 (Figure 1).

## DISCUSSION

### Overview of antimicrobial resistance and virulence in *K. pneumoniae*

This study presents evidence of high antimicrobial resistance and prevalence of virulence genes in *K. pneumoniae* isolates from both domestic animals and humans in the state of Mato Grosso, Brazil. The detection of MDR strains – particularly those harboring clinically relevant STs such as ST15, ST11, and ST437 – highlights the risk of zoonotic transmission and the convergence of resistance and virulence traits. Our findings underscore the need for integrated surveillance strategies within the One Health framework, particularly in regions where data are scarce.

### MDR prevalence and infection types

The MDR *K. pneumoniae* primarily causes urinary, enteric, and respiratory infections, consistent with findings reported in previous studies by Diancourt *et al*. [24] and Cardoso Almeida *et al*. [25]. In this study, MDR *K. pneumoniae* was detected in 100% of animal isolates and 53% of human isolates. This highlights a major challenge for One Health initiatives – the transmission of MDR pathogens through direct human–animal contact and through contaminated environments [1, 26]. Animals are of particular concern as reservoirs and vectors for the dissemination of antimicrobial resistance genes. This issue is especially pertinent in veterinary medicine, as prior research by Ewers *et al*. [27] has underscored the significant role of *K. pneumoniae* in nosocomial infections.

### Resistance to β-lactams and carbapenems

*K. pneumoniae* exhibited the highest resistance to β-lactam antibiotics [28, 29], with intrinsic resistance to ampicillin and penicillin confirmed in isolates from sheep and goats in Egypt. Carbapenem resistance remains relatively rare in veterinary medicine, largely due to the limited and judicious use of this antibiotic class, which is typically reserved for severe infections in companion animals. Nevertheless, reports from other countries indicate a rising trend in carbapenem resistance among animal-derived isolates [30–32]. Although *bla*_KPC-2_ has been identified in *K. quasipneumoniae* isolates in Brazil, no such findings have been reported in animal-derived *K. pneumoniae* isolates to date [30–32]. Human isolates often exhibit elevated resistance levels due to the expression of carbapenemase enzymes, particularly KPC [33, 34]. Additionally, a study from Egypt revealed high resistance to penicillins, cephalosporins, and sulfonamides among *K. pneumoniae* isolates from sheep and goats [35].

### Virulence determinants and their functions

To establish infection, *K. pneumoniae* must circumvent mechanical and chemical barriers and evade innate immune responses [36]. The virulence of *K. pneumoniae* is multifactorial, involving various genetic and structural determinants. Fimbrial adhesins, such as *fimH* and *mrkD*, facilitate bacterial adher-ence to the urinary epithelium, thereby enhancing colonization [36]. Other virulence factors, such as *ugE* and *wabG*, aid in evasion of the complement system, whereas *entB* and *kfu* contribute to iron acquisition and bacterial proliferation [37–39].

### Prevalence of virulence genes in human and animal isolates

The prevalence of virulence genes in this study aligns with findings by Kot *et al*. [37] and Kus *et al*. [40], who reported high detection rates of *entB, mrkD*, and *fimH* in human isolates. Numerous veterinary studies have characterized virulence genes in MDR *K. pneumoniae* isolates from animals [41–43]. Consistent with our findings, these investigations also reported frequent detection of *wabG, entB*, and *fimH* among animal isolates. In the current study, *entB* was the most prevalent virulence gene detected in both animal and human isolates. This observation is consistent with Davoudabadi *et al*. [44], who reported 100% prevalence of *entB* in 52 human *K. pneumoniae* isolates. The findings confirm the presence of multiple virulence factors, including *entB, ugE, fimH, wabG*, and *mrkD*, in fecal samples from diverse host species. This is in agreement with Amaretti *et al*. [45], who also reported high prevalence of *entB* and *mrkD* in *K. pneumoniae* isolates from human fecal samples. Contrary to Osman *et al*. [41], who detected high prevalence of *kfu* and *ugE* genes in buffalo milk samples with mastitis, this study found no virulence genes in bovine milk-derived isolates. Nonetheless, these findings should be interpreted cautiously due to the limited number of samples associated with mastitis.

### Role of *fimH* in urinary infections

The *fimH* gene, encoding fimbrial adhesins, was identified in 43.75% (21/48) of total isolates and in 23.80% (5/21) of those from urine samples. Previous studies by Jiang *et al*. [46] and Sarshar *et al*. [47] have highlighted the critical role of *fimH* in urinary tract infections caused by *Enterobacteriaceae*.

### Genetic diversity and ST distribution

Genetic analysis revealed substantial population diversity among *K. pneumoniae* isolates in this study. The isolates demonstrated broad ST variability, including several newly identified STs and singletons. Despite ST diversity, high nucleotide identity among isolates indicated potential clonal similarity between animal- and human-derived strains [26, 48–51]. Notably, ST15 and ST307 identified here are epidemiologically significant due to their established association with nosocomial infections. These STs have been described in Japan as public health concerns due to their widespread association with hospitals [49]. In Portugal, ST15 has been implicated in both community- and hospital-acquired infections, including those in intensive care settings [52]. ST15 has also been reported in domestic animals across multiple European countries – including Portugal, Germany, France, and others – where it is associated with diverse clinical manifestations [27]. In this study, ST15 isolated from canine urine was found to express multiple virulence genes, including *fimH*, *mrkD*, *entB*, *ugE*, and *kfu*. In Brazil, ST15 has also been identified in polluted urban waterways, with genomic similarity to ST15 strains from humans and animals, including shared virulence factors [53]. ST15 harbors genomic traits that may enhance its dissemination and is increasingly recognized as a biomarker for the surveillance of high-risk *K. pneumoniae* clones [54].

### High-risk CCs and global spread

Additional human isolates included ST11 and ST437, both members of the high-risk clonal complex CC258. The same STs were observed in human isolates from Brazil [33]. A 4-year retrospective study in China found ST11 to be the predominant strain among patients with carbapenem-resistant *K. pneumoniae*, and it was associated with a high 30-day mortality rate [55]. The ST11 lineage poses a substantial global health threat due to its strong association with hospital outbreaks and extensive dissemination [55, 56]. Furthermore, ST11 has been reported in animal hosts across Germany, Italy, Switzerland, Taiwan, and Japan [28, 57–59]. ST35, isolated from a dog with otitis, has been previously identified in various sources, including pork meat, nosocomial infections, environmental infections, bovine milk samples, and wild animals [60–62]. Animal isolates raise great concern because they can colonize humans and contaminate the environment [30]. Hence, measures must be adopted to prevent the spread of high-risk clonal lineages within the population [31].

### Zoonotic potential of CC147 and cross-species transmission

As observed in the human and canine isolates, the expression of CC147 was similar to that observed in northern Brazil. This clonal complex is commonly found in hospital outbreaks, having been reported in Greece and Italy [7, 63, 64]. This finding highlights the possibility of clonal dispersal between different species, as reported by Marques *et al*. [31], who documented fecal colonization by *K. pneumoniae* in companion animals and healthy humans sharing similar clonal lineages [31].

### Drivers of resistance and need for control measures

The increase in resistance to antibiotics is mainly due to their intensive and inappropriate use, combined with the inherent ability of bacteria to mutate, which favors the selection of resistant strains [2]. Consequently, antibiotics lose their effectiveness, leading to thera-peutic failure, increased healthcare costs, and even death in extreme cases [5]. Healthcare professionals, producers, health authorities, and pharmaceutical companies must implement appropriate measures to reduce the transmission of resistance to mitigate this problem. This effort requires careful consideration of bacterial epidemiology, human–animal interactions, the appropriate use of antimicrobials in all species, adherence to general infection control principles, and the implementation of appropriate public health hygiene measures to reduce transmission. To control antimicrobial resistance, modern animal husbandry and slaughter practices should be adopted, and proper handling and preparation of food should be ensured [64, 65].

### Study limitations and regional relevance

Although our study has limitations, such as the relatively small sample size (48 isolates) and the unequal distribution between domestic animals (69%) and humans (31%), the data obtained are unprecedented for the state of Mato Grosso. This contribution becomes even more relevant given the scarcity of research on the resistance and virulence profiles of *K. pneumoniae* in this regional context. While the predominance of samples from hospital and clinical environments may reflect more severe cases, the findings provide valuable insights and establish a crucial foundation for future, more comprehensive and targeted investigations, thereby strengthening control and prevention strategies within the One Health framework. In this context, it is important to highlight that the samples were collected between 2016 and 2017. Despite the temporal gap, the data remain epidemiologically relevant due to the absence of similar regional studies. Thus, this work fills a critical knowledge gap and offers a strategic baseline for future monitoring and intervention efforts in both human and animal health.

## CONCLUSION

This study provides the first integrated molecular epidemiological assessment of *K. pneumoniae* isolates from domestic animals and humans in the state of Mato Grosso, Brazil, reinforcing the importance of One Health-based surveillance. Among the 48 isolates analyzed, a high overall prevalence of multidrug resistance was observed, at 78.78% in animal isolates and 53% in human isolates, resulting in a total MDR rate of 70.83%. Notably, the *bla*_KPC-2_ gene was exclusively detected in human isolates (20%), while *bla*_NDM_ was found in both human (6.66%) and bovine (3.03%) isolates, reflecting potential interspecies exchange of critical resistance determinants. Virulence gene screening revealed that 77.08% of isolates harbored at least one virulence factor, with *entB*, *fimH*, and *wabG* being the most frequent, suggesting a convergence of resistance and pathogenicity traits in both hosts.

MLST analysis unveiled substantial genetic diversity, with 39 distinct STs, including nine novel STs, and the presence of CCs such as CC258 (human), CC15 (animal), and the interspecies-shared CC147. These findings underscore the presence of high-risk clones capable of crossing species barriers, highlighting the zoonotic threat posed by companion and production animals in close contact with humans. The results call for immediate implementation of integrated antimicrobial stewardship and biosecurity policies across veterinary and human healthcare sectors. Diagnostic laboratories should prioritize the use of molecular tools, such as MLST and resistance gene screening, for rapid detection of high-risk *K. pneumoniae* strains. Furthermore, veter-inary practitioners should be cautious with empirical antimicrobial usage, especially with β-lactams and sulfonamides, given their high resistance rates in animal isolates.

A major strength of this study is the dual-host, dual-environment design that bridges human and veterinary microbiology under the One Health para-digm. The use of comprehensive molecular typing and resistance profiling tools provides robust genotypic evidence to support surveillance and control strategies. The identification of novel STs contributes new data to global MLST databases, enhancing the scientific community’s capacity to trace transmission pathways. Given the limited regional data and small sample size, future studies should expand longitudinal sampling across diverse geographic areas and ecological settings. Whole-genome sequencing and plasmid analysis will further clarify horizontal gene transfer events and reveal additional mechanisms of resistance. Environmental sampling from shared human-animal ecosystems (e.g., water sources, animal shelters, and farms) would also enrich our understanding of reservoirs and transmission dynamics. In summary, the study highlights the crit-ical need for harmonized antimicrobial resistance monitoring across the animal–human interface in Brazil. The identification of shared virulence traits and MDR phenotypes in genetically related *K. pneumoniae* clones emphasizes the importance of coordinated surveillance, timely diagnostics, and intersectoral collaboration to mitigate the public health threat posed by high-risk clones. These findings contribute a foundational dataset for regional and national policymakers aiming to strengthen One Health-informed AMR control efforts.

## AUTHORS’ CONTRIBUTIONS

ATHIS, LN, and VD: Conceptualized and designed the study and drafted the manuscript. HM, MTSC, SLC, KLTG, CSC, MAP, FKSFA, FJDS, LN, and VD: Conducted research, collected samples, and performed laboratory work. ATHIS, HM, MTSC, SLC, KLTG, CSC, MAP, FKSFA, FJDS, LN, and VD: Analyzed and interpreted the data. ABPFA, VRFS, LN, and VD: Supervised the project and revised the manuscript. All authors have read and approved the final manuscript.

## References

[1] Wareth G, Neubauer H (2021). The Animal-foods-environment interface of *Klebsiella pneumoniae* in Germany:An observational study on pathogenicity, resistance development and the current situation. Vet. Res.

[2] World Health Organization (2024). Antimicrobial Resistance:Accelerating National and Global Responses:Report of the 77^th^ World Health Assembly (WHA77).

[3] Joseph B.J, Mathew M, Rachel R, Mathew J, Radhakrishnan E.K, Busi S, Prasad R (2024). *Klebsiella pneumoniae* virulence factors and biofilm components:Synthesis, structure, function, and inhibitors. ESKAPE Pathogens.

[4] Monteiro A.S.S, Cordeiro S.M, Reis J.N (2024). Virulence factors in *Klebsiella pneumoniae*:A literature review. Indian J. Microbiol.

[5] World Health Organization (WHO) (2023). Antibiotic Resistance.

[6] Yao Y, Zha Z, Li L, Tan H, Pi J, You C, Liu B (2024). Healthcare-associated carbapenem-resistant *Klebsiella pneumoniae* infections are associated with higher mortality compared to carbapenem-susceptible *K. pneumoniae* infections in the intensive care unit:A retrospective cohort study. J. Hosp. Infect.

[7] Rodrigues Y.C, Lobato A.R.F, Quaresma A.J.P.G, Guerra L.M.G.D, Brasiliense D.M (2021). The spread of NDM-1 and NDM-7-producing *Klebsiella pneumoniae* is driven by multiclonal expansion of high-risk clones in healthcare institutions in the state of Pará, Brazilian Amazon Region. Antibiotics (Basel).

[8] Xu Q, Xie M, Tang Y, Heng H, Yang X, Liu X, Chan E.W.C, Yang G, Chen S (2024). Enhancing resistance, but not virulence attributed to the high mortality caused by carbapenem-resistant *Klebsiella pneumoniae*. Microbiol. Res.

[9] Quinn P.J, Carter M.E, Markey B.K (2013). General procedures in microbiology. In:Clinical Veterinary Microbiology.

[10] Bauer A.W, Kirby W.M, Sherris J.C, Turck M (1966). Antibiotic susceptibility testing by a standardized single disk method. Am. J. Clin. Pathol.

[11] CLSI (2023). Performance Standards for Antimicrobial Disk and Dilution Susceptibility Tests for Bacteria Isolated from Animals. CLSI supplement VET01S.

[12] CLSI (2023). Performance Standards for Antimicrobial Susceptibility Testing. CLSI Supplement M100.

[13] BrCAST-Brazilian Committee on Antimicrobial Susceptibility Testing (BrCAST /EUCAST) (2023). Tables of Cut-off Points for Interpreting MICs and Halo Diameters - Version in Portuguese of the EUSCAST Breakpoint Tables for Interpretation of MICs and Zone Diameters.

[14] Magiorakos A.P, Srinivasan A, Carey R.B, Carmeli Y, Falagas M.E, Giske C.G, Paterson D.L, Rice L.B, Stelling J, Struelens M.J, Vatopoulos A, Weber J.T, Monnet D.L (2012). Multidrug-resistant, extensively drug-resistant and Pandrug-resistant bacteria:An international expert proposal for interim standard definitions for acquired resistance. Clin. Microbiol. Infect.

[15] Sambrook J, Russell D.W (2004). Molecular Cloning:A Laboratory Manual.

[16] Lane D.J (1991). 16S/23S rRNA sequencing. In:Nucleic Acid Techniques in Bacterial Systematics.

[17] Turner S, Pryer K.M, Miao V.P.W, Palmer J.D (1999). Investigating deep phylogenetic relationships among cyanobacteria and plastids by small subunit rRNA sequence analysis. J. Eukaryot. Microbiol.

[18] Brisse S, Grimont F, Grimont P, Dworkin M, Falkow S, Rosenberg E, Schleifer K.H, Stackebrandt E (1999). The genus *Klebsiella*. The Prokaryotes:A Handbook on the Biology of Bacteria.

[19] Regue M, Hita B, Pique N, Izquierdo L, Merino S, Fresno S (2004). The gene, Uge, is essential for *Klebsiella pneumoniae* virulence. Infect. Immunity.

[20] Lafeuille E, Decre D, Mahjoub-Messai F, Bidet P, Arlet G, Birgen E (2013). OXA-48 carbapenemase-producing *Klebsiella pneumoniae* isolated from Libyan patients. Microbial Drug Resist.

[21] Ma L.C, Fang C.T, Lee C.Z, Shun C.T, Wang J.T (2005). Genomic heterogeneity in *Klebsiella pneumoniae* strains is associated with primary pyogenic liver abscess and metastatic infection. J. Infect. Dis.

[22] Yigit H, Queenan A.M, Anderson G.J, Domenech-Sanchez A, Biddle J.W, Steward C.D, Alberti S, Bush K, Tenover F.C (2001). Novel carbapenem-hydrolyzing β-lactamase, KPC-1, from a carbapenem-resistant strain of *Klebsiella pneumoniae*. Antimicrob. Agents Chemother.

[23] Feng Y, Xue G, Feng J, Yan C, Cui J, Gan L, Zhang R, Zhao H, Xu W, Li N, Liu S, Du S, Zhang W, Yao H, Tai J, Ma L, Zhang T, Qu D, Wei Y, Yuan J (2021). Rapid detection of New Delhi metallo-β-lactamase gene using recombinase-aided amplification directly on clinical samples from children. Front. Microbiol.

[24] Diancourt L, Passet V, Verhoef J, Grimont P.A, Brisse S (2005). Multilocus sequence typing of *Klebsiella pneumoniae* nosocomial isolates. J. Clin. Microbiol.

[25] Cardoso Almeida A.P, de Moraes M.A, da Silva A.K.F, Oliveira-Silva M, Nakamura-Silva R, de Almeida F.M, Pappas Junior G.J, Pitondo-Silva A, de Campos T.A (2024). Long-term occurrence of multiple antimicrobial drug resistant *Klebsiella pneumoniae* isolates harboring virulent potential in a tertiary hospital from Brazil. Braz. J. Microbiol.

[26] Wyres K.L, Holt K.E (2016). *Klebsiella pneumoniae* population genomics and antimicrobial-resistant clones. Trends Microbiol.

[27] Ewers C, Stamm I, Pfeifer Y, Wieler L.H, Kopp P.A, Schønning K (2014). Clonal spread of highly successful ST15-CTX-M-15 *Klebsiella pneumoniae* in companion animals and horses. J. Antimicrob. Chemother.

[28] Ovejero C.M, Escudero J.A, Thomas-Lopez D, Hoefer A, Moyano G, Montero N, Gonzalez-Zorn B (2017). Highly tigecycline-resistant *Klebsiella pneumoniae* sequence type 11 (ST11) and ST147 isolates from companion animals. Antimicrob. Agents Chemother.

[29] Frenzer S.K, Feuer L, Bäumer W, Lübke-Becker A, Klein B, Merle R (2024). Temporal trends in antimicrobial resistance of *Klebsiella pneumonia* in clinically affected canine and feline populations in Germany:A 2019-2021 analysis. Microbiol. Res.

[30] Davis G.S, Waits K, Nordstrom L, Weaver B, Aziz M, Gauld L, Stegger M (2015). Intermingled *Klebsiella pneumoniae* populations between retail meats and human urinary tract infections. Clin. Infect. Dis.

[31] Marques C, Belas A, Aboim C, Cavaco-Silva P, Trigueiro G, Gama L.T, Pomba C (2019). Evidence of sharing of *Klebsiella pneumoniae* strains between healthy companion animals and cohabiting humans. J. Clin. Microbiol.

[32] Sellera F.P, Da Silva L.C, Lincopan N (2021). Rapid spread of critical priority carbapenemase-producing pathogens in companion animals:A One Health challenge for a post-pandemic world. J. Antimicrob. Chemother.

[33] Pereira P.S, Borghi M, de Araújo C.F, Aires C.A, Oliveira J.C, Asensi M.D, Assef A.P (2015). Clonal dissemination of OXA-370-producing *Klebsiella pneumoniae* in Rio de Janeiro, Brazil. Antimicrob. Agents Chemother.

[34] Jiang J, Long T, Porter A.R, Lovey A, Lee A, Jacob J.T, Arias C.A, Bonomo R.A, Kalayjian R, Zhao Y, DeLeo F.R, van Duin D, Kreiswirth B.N, Chen L (2025). Carbapenem-resistant, virulence plasmid-harboring *Klebsiella pneumoniae*, United States. Emerg. Infect. Dis.

[35] Mahrous S.H, El-Balkemy F.A, Abo-Zeid N.Z, El-Mekkawy M.F, El Damaty H.M, Elsohaby I (2023). Antibacterial and anti-biofilm activities of cinnamon oil against multidrug-resistant *Klebsiella pneumoniae* isolated from pneumonic sheep and Goats. Pathogens.

[36] Hu Y.L, Bi S.L, Zhang Z.Y, Kong N.Q (2024). Correlação entre resistência a antibióticos, genes de virulência e genótipos entre cepas clínicas de *Klebsiella pneumoniae* isoladas em Guangzhou, China. Curr. Microbiol.

[37] Kot B, Piechota M, Szweda P, Mitrus J, Wicha J, Grużewska A, Witeska M (2023). Virulence analysis and antibiotic resistance of *Klebsiella pneumoniae* isolates from hospitalized patients in Poland. Sci. Rep.

[38] Lee C.R, Lee J.H, Park K.S, Jeon J.H, Kim Y.B, Cha C.J, Jeong B.C, Lee S.H (2017). Antimicrobial resistance of hypervirulent *Klebsiella pneu-moniae*:Epidemiology, hypervirulence-associated determinants, and resistance mechanisms. Front. Cell. Infect. Microbiol.

[39] Runci F, Gentile V, Frangipani E, Rampioni G, Leoni L, Lucidi M, Visaggio D, Harris G, Chen W, Stahl J, Averhoff B, Visca P (2019). Contribution of active iron uptake to *Acinetobacter baumannii* pathogenicity. Infect. Immunity.

[40] Kus H, Arslan U, Türk H.D, Fındık D (2017). Investigation of various virulence factors of *Klebsiella pneumoniae* strains isolated from nosocomial infections. Mikrobiyol Bul.

[41] Osman K.M, Hassan H.M, Orabi A, Abdelhafez A.S (2014). Phenotypic, antimicrobial susceptibility profile and virulence factors of *Klebsiella pneumoniae* isolated from buffalo and cow mastitic milk. Pathog. Glob. Health.

[42] Yang F, Deng B, Liao W, Wang P, Chen P, Wei J (2019). High rate of multiresistant *Klebsiella pneumoniae* from human and animal origin. Infect. Drug Resist.

[43] Hossain S, De Silva B.C.J, Dahanayake P.S, Heo G.J (2020). Phylogenetic relationships, virulence and antimicrobial resistance properties of *Klebsiella* sp. isolated from pet turtles in Korea. Lett. Appl. Microbiol.

[44] Davoudabadi S, Goudarzi M, Hashemi A (2023). Detection of virulence factors and antibiotic resistance among *Klebsiella pneumoniae* isolates from Iran. BioMed Res. Int.

[45] Amaretti A, Righini L, Candeliere F, Musmeci E, Bonvicini F, Gentilomi G.A, Raimondi S (2020). Antibiotic resistance, virulence factors, phenotyping, and genotyping of non-*Escherichia coli* Enterobacterales from the gut microbiota of healthy subjects. Int. J. Mol. Sci.

[46] Jiang W, Chen Y, Lai M, Ji Y, Lin S, Shao J, Chen X (2025). Comprehensive genomic epidemiology and antimicrobial resistance profiles of clinical *Klebsiella pneumoniae* species complex isolates from a tertiary hospital in Wenzhou, China (2019–2021). BMC Genomics.

[47] Sarshar M, Behzadi P, Ambrosi C, Zagaglia C, Palamara A.T, Scribano D (2020). FimH and anti-adhesive therapeutics:A disarming strategy against uropathogens. Antibiotics.

[48] Holt K.E, Wertheim H, Zadoks R.N, Baker S, Whitehouse C.A, Dance D, Brisse S (2015). Genomic analysis of diversity, population structure, virulence, and antimicrobial resistance in *Klebsiella pneumoniae*, an urgent threat to public health. Proc. Natl. Acad. Sci.

[49] Harada K, Shimizu T, Mukai Y, Kuwajima K, Sato T, Usui M, Ohki A (2016). Phenotypic and molecular characterization of antimicrobial resistance in *Klebsiella* spp. isolates from companion animals in Japan:Clonal dissemination of multidrug-resistant extended-spectrum β-lactamase-producing *Klebsiella pneumoniae*. Front. Microbiol.

[50] Maeyama Y, Taniguchi Y, Hayashi W, Ohsaki Y, Osaka S, Koide S, Nagano N (2018). Prevalence of ESBL/ AmpC genes and specific clones among the third-generation cephalosporin-resistant Enterobacteriaceae from canine and feline clinical specimens in Japan. Vet. Microbiol.

[51] Wyres K.L, Lam M, Holt K.E (2020). Population genomics of *Klebsiella pneumoniae*. Nat. Rev. Microbiol.

[52] Rodrigues C, Machado E, Ramos H, Peixe L, Novais Â (2014). Expansion of ESBL-producing *Klebsiella pneumoniae* in hospitalized patients:A successful story of international clones (ST15, ST147, ST336) and epidemic plasmids (IncR, IncFIIK). Int. J. Med. Microbiol.

[53] Cardoso B, Esposito F, Fontana H, Fuga B, Moura Q, Sano E, Sato M.I.Z, Brandão C.J, Oliveira F.A, Levy C.E, Huenuman N.E, Lincopan N (2022). Genomic analysis of a Kpi (pilus system)-positive and CTX-M-15-producing *Klebsiella pneumoniae* belonging to the high-risk clone ST15 isolated from an impacted river in Brazil. Genomics.

[54] Gato E, Rodiño-Janeiro B.K, Gude M.J, Fernández-Cuenca F, Pascual A, Fernández A, &Bou G (2023). Diagnostic tool for surveillance, detection and monitoring of the high-risk clone *K. pneumoniae* ST15. J. Hosp. Infect.

[55] Cheng J, Zhao D, Ma X, Li J (2023). Molecular epidemiology, risk factors, and outcomes of carbapenem-resistant *Klebsiella pneumoniae* infection in a tertiary hospital in eastern China:For a retrospective study conducted over 4 years. Front. Microbiol.

[56] Marques C, Menezes J, Belas A, Aboim C, Cavaco-Silva P, Trigueiro G, Pomba C (2018). *Klebsiella pneumoniae* causing urinary tract infections in companion animals and humans:Population structure, antimicrobial resistance and virulence genes. J. Antimicrob. Chemother.

[57] Hidalgo L, Gutierrez B, Ovejero C.M, Carrilero L, Matrat S, Saba C.K, Santurde G (2013). *Klebsiella pneumoniae* sequence type 11 from companion animals bearing ArmA methyltransferase, DHA-1 β-lactamase, and QnrB4. Antimicrob. Agents Chemother.

[58] Zog A.L, Simmen S, Zurfluh K, Stephan R, Schmitt S.N, Nüesch-Inderbinen M (2018). High prevalence of extended-spectrum β-lactamase producing Enterobacteriaceae among clinical isolates from cats and dogs admitted to a veterinary hospital in Switzerland. Front. Vet. Sci.

[59] Pulss S, Stolle I, Stamm I, Leidner U, Heydel C, Semmler T, Ewers C (2018). Multispecies and clonal dissemination of OXA-48 carbapenemase in Enterobacteriaceae from companion animals in Germany, 2009-2016. Front. Microbiol.

[60] Bialek-Davenet S, Criscuolo A, Ailloud F, Passet V, Jones L, Delannoy-Vieillard A.S, Garin B, Le Hello S, Arlet G, Nicolas-Chanoine M.H, Decré D, Brisse S (2014). Genomic definition of hypervirulent and multidrug-resistant *Klebsiella pneumoniae* clonal groups. Emerg. Infect. Dis.

[61] Wang J, Zeng Z.L, Huang X.Y, Ma Z.B, Guo Z.W, Lv L.C, Liu J.H (2018). Evolution and comparative genomics of F33:A?:B? plasmids carrying *bla*_CTX-M-55_ or *bla*_CTX-M-65_ in *Escherichia coli* and *Klebsiella pneumoniae* isolated from animals, food products, and humans in China. Msphere.

[62] Cornacchia A, Chiaverini A, Centorotola G, Di Domenico M, Cocco A, Ancora M, Cammà C, D'Alterio N, Di Francesco C.E, Pomilio F (2022). Whole-genome sequences of two *Klebsiella pneumoniae* strains (sequence types 23 and 35) from wildlife. Microbiol. Resour. Announc.

[63] Hasan C.M, Turlej-Rogacka A, Vatopoulos A.C, Giakkoupi P, Maatallah M, Giske C.G (2014). Dissemination of *bla*_VIM_ in Greece at the peak of the epidemic of 2005–2006:Clonal expansion of *Klebsiella pneumoniae* clonal complex 147. Clin. Microbiol. Infect.

[64] Mileto I, Petazzoni G, Corbella M, Gaiarsa S, Merla C, Kuka A, Ramus M, Terulla C, Brandolini M, Piralla A, Cambieri P, Baldanti F (2023). Rapid spread of a novel NDM-producing clone of *Klebsiella pneumoniae* CC147, Northern Italy, February to August 2023. Eurosurveillance.

[65] Ndlovu T, Kgosietsile L, Motshwarakgole P, Ndlovu S.I (2023). Evaluation of potential factors influencing the dissemination of multidrug-resistant *Klebsiella pneumoniae* and alternative treatment strategies. Trop. Med. Infect. Dis.

